# Supplementary material for: High Levels of Progesterone Receptor B in MCF-7 Cells Enable Radical Anti-Tumoral and Anti-Estrogenic Effect of Progestin
Source: Biomedicines. 2022 Aug 2;10(8):1860. doi: 10.3390/biomedicines10081860 (PMC9405688; doi:10.3390/biomedicines10081860)
Supplement: Supplementary file 1 [file biomedicines-10-01860-s001.zip › Supplementary Figure S1.pdf]

A

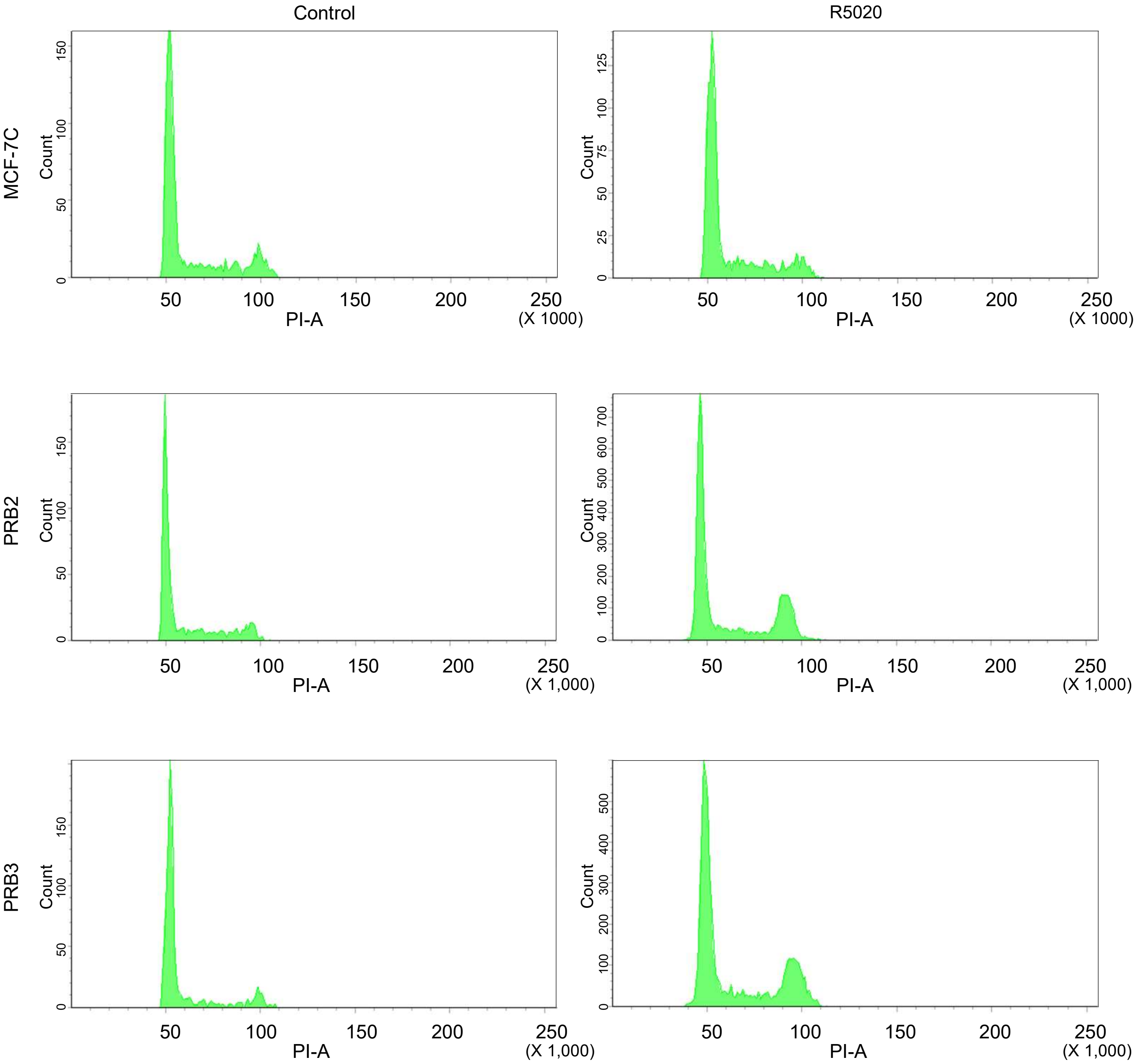

B

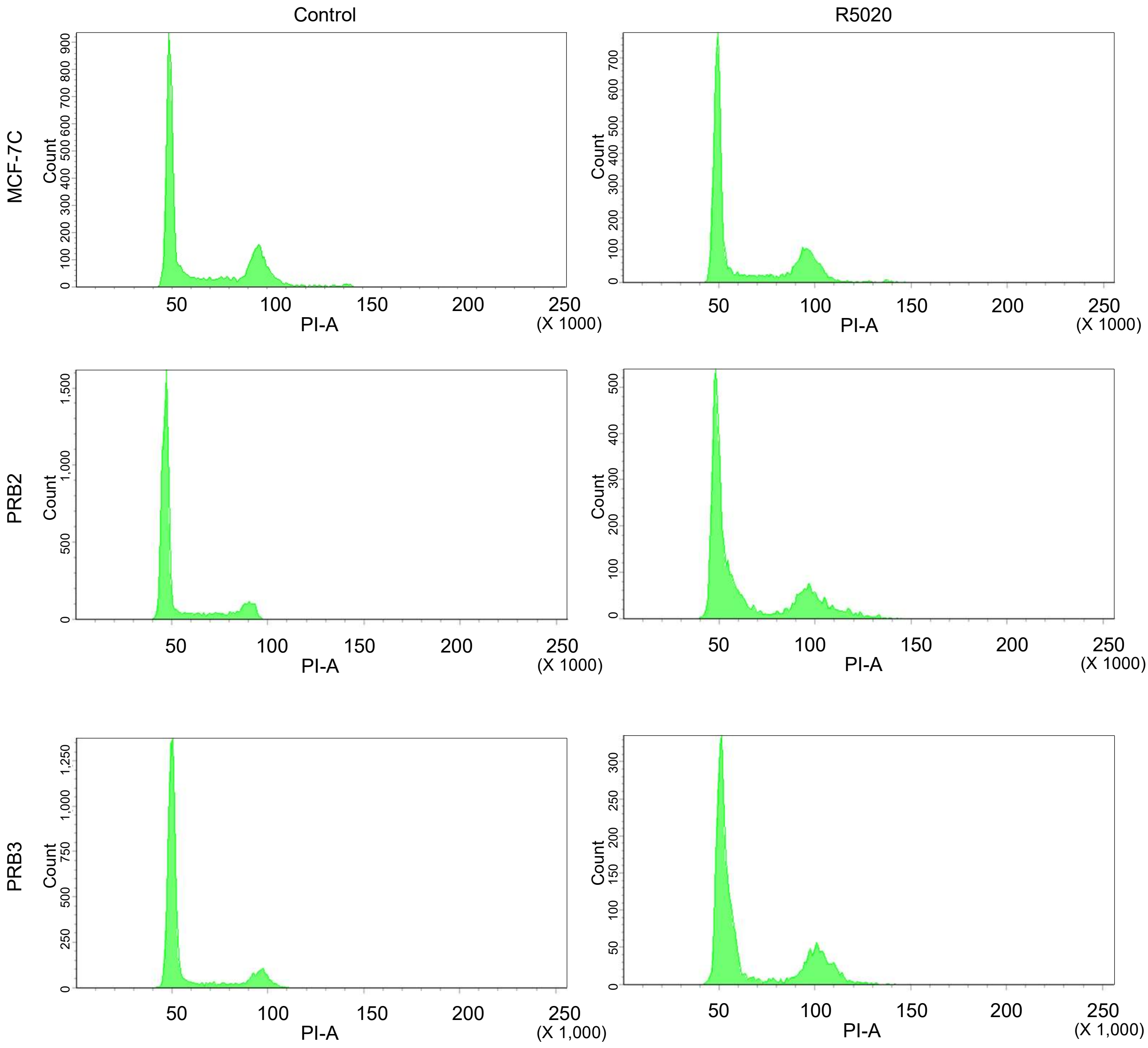

Supplementary Figure S1. Representative histograms of cell cycle distribution of MCF-7C and PRB2, PRB3 cells using the program ModFit-LT. **A.** Cell cycle distribution after 24 hours treatment with control vehicle or R5020. R5020 causes an increase in cells in the s phase and G2M phase. But the data is only significant in PRB2 cells. **B.** Cell cycle distribution after 96 hours treatment with control vehicle or R5020. The histograms show a decrease of s phase cells and an accumulation of G2/M cells after R5020-treatment of PRB2 and PRB3.
