# Supplementary material for: High Levels of Progesterone Receptor B in MCF-7 Cells Enable Radical Anti-Tumoral and Anti-Estrogenic Effect of Progestin
Source: Biomedicines. 2022 Aug 2;10(8):1860. doi: 10.3390/biomedicines10081860 (PMC9405688; doi:10.3390/biomedicines10081860)
Supplement: Supplementary file 1 [file biomedicines-10-01860-s001.zip › Supplementary Figure S2.pdf]

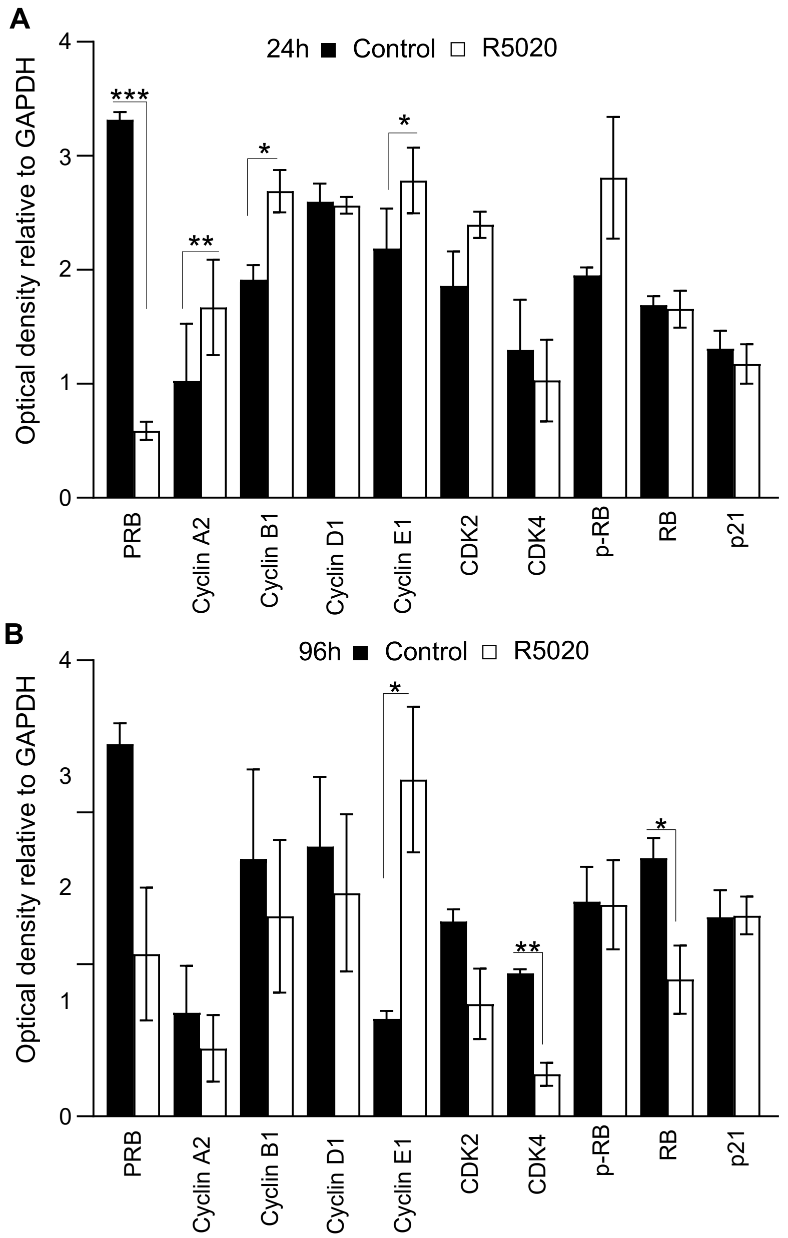

Supplementary Figure S2. Densitometry analysis of the Western blots in Figure 2A. The optical density were quantitated by ImageJ. The results are expressed relative to the intensity of GAPDH for each sample. The data are from two independent experiments with PRB2 and PRB3 considered as individual observation. There is therefore 4 data points in each group. The results are expressed as mean ± SEM (n=4). There is a large variation of optical density in the two independent experiments due to exposure time etc, leading to large error bars and lack of statistical significance in the case of CDK2, which looks obviously decreased in R5020-treated cells in individual experiments. GAPDH was used as a loading control.
